# Precursor-Boosted Production of Metabolites in *Nasturtium officinale* Microshoots Grown in Plantform Bioreactors, and Antioxidant and Antimicrobial Activities of Biomass Extracts

**DOI:** 10.3390/molecules26154660

**Published:** 2021-07-31

**Authors:** Marta Klimek-Szczykutowicz, Michał Dziurka, Ivica Blažević, Azra Đulović, Małgorzata Miazga-Karska, Katarzyna Klimek, Halina Ekiert, Agnieszka Szopa

**Affiliations:** 1 Chair and Department of Pharmaceutical Botany, Faculty of Pharmacy, Medical College, Jagiellonian University, Medyczna 9, 30-688 Kraków, Poland; marta.klimek-szczykutowicz@doctoral.uj.edu.pl (M.K.-S.); halina.ekiert@uj.edu.pl (H.E.); 2 Department of Dermatology, Cosmetology and Aesthetic Surgery, The Institute of Medical Sciences, Medical College, Jan Kochanowski University, Stefana Żeromskiego 5, 25-369 Kielce, Poland; 3 The Franciszek Górski Institute of Plant Physiology, Polish Academy of Sciences, Niezapominajek 21, 30-239 Kraków, Poland; m.dziurka@ifr-pan.edu.pl; 4 Department of Organic Chemistry, Faculty of Chemistry and Technology, University of Split, Ruđera Boškovića 35, 21000 Split, Croatia; blazevic@ktf-split.hr (I.B.); azra@ktf-split.hr (A.Đ.); 5 Chair and Department of Biochemistry and Biotechnology, Faculty of Pharmacy, Medical University of Lublin, Chodźki 1, 20-046 Lublin, Poland; malgorzata.miazga-karska@umlub.pl (M.M.-K.); katarzyna.klimek@umlub.pl (K.K.)

**Keywords:** in vitro cultures, Plantform bioreactor, L-phenylalanine, L-tryptophan, glucosinolates, polyphenol compounds, photosynthetic pigments, saccharides, antioxidant activity, antimicrobial activity

## Abstract

The study demonstrated the effects of precursor feeding on the production of glucosinolates (GSLs), flavonoids, polyphenols, saccharides, and photosynthetic pigments in *Nasturtium officinale* microshoot cultures grown in Plantform bioreactors. It also evaluated the antioxidant and antimicrobial activities of extracts. L-phenylalanine (Phe) and L-tryptophan (Trp) as precursors were tested at 0.05, 0.1, 0.5, 1.0, and 3.0 mM. They were added at the beginning (day 0) or on day 10 of the culture. Microshoots were harvested after 20 days. Microshoots treated with 3.0 mM Phe (day 0) had the highest total GSL content (269.20 mg/100 g DW). The qualitative and quantitative profiles of the GSLs (UHPLC-DAD-MS/MS) were influenced by precursor feeding. Phe at 3.0 mM stimulated the best production of 4-methoxyglucobrassicin (149.99 mg/100 g DW) and gluconasturtiin (36.17 mg/100 g DW). Total flavonoids increased to a maximum of 1364.38 mg/100 g DW with 3.0 mM Phe (day 0), and polyphenols to a maximum of 1062.76 mg/100 g DW with 3.0 mM Trp (day 0). The precursors also increased the amounts of *p*-coumaric and ferulic acids, and rutoside, and generally increased the production of active photosynthetic pigments. Antioxidant potential increased the most with 0.1 mM Phe (day 0) (CUPRAC, FRAP), and with 0.5 mM Trp (day 10) (DPPH). The extracts of microshoots treated with 3.0 mM Phe (day 0) showed the most promising bacteriostatic activity against microaerobic Gram-positive acne strains (MIC 250–500 µg/mL, 20–21 mm inhibition zones). No extract was cytotoxic to normal human fibroblasts over the tested concentration range (up to 250 μg/mL).

## 1. Introduction

*Nasturtium officinale* R. Br (Brassicaceae) is increasingly known for its health properties, as well as its use as a raw material in “superfood” or “fit food” [1]. In its natural environment, it grows near streams, rivers, and wetlands. *N. officinale* belongs to leafy vegetables and is grown mostly in hydroponic farms. This species is characterized by a rapid increase in biomass; after 4–8 weeks the plants are ready for harvesting [2,3]. The European Food Safety Authority (EFSA) has classified *N. officinale* as a safe vegetable under the group “Leaf vegetables, herbs and edible flowers” [4]. *N. officinale* possesses a low calorific value (11 calories in 100 g of FW (fresh weight)), and is also a rich source of valuable compounds such as glucosinolates (GSLs), isothiocyanates, polyphenols, vitamins (B_1_, B_2_, B_3_, B_6_, E, C), and bioelements [5,6,7,8]. Due to its biological activity and the characteristic flavor caused by isothiocyanates, it is often added to dishes in, e.g., European countries, Brazil, and the USA [9]. In Europe, this species has been classified by the International Union for Conservation of Nature (IUCN) Red List of Threatened Species differently in each European country; for example, it is considered an extinct or probably extinct plant in Estonia, endangered in Austria and Sweden, and partly endangered in Poland.

The main group of bioactive compounds in the herb of *N. officinale* are GSLs, namely glucobrassicin, 4-hydroxyglucobrassicin, 4-methoxyglucobrassicin (indole GSLs), and gluconasturtiin (aromatic GSL). After natural hydrolysis by myrosinase enzymes, GSLs cause the formation of isothiocyanates [9]. Studies of these compounds have confirmed their protective activity against cancer [10,11,12]. Beneficial effects of extracts of *N. officinale herb* have also been scientifically proven, e.g., their antioxidant, antibacterial, and anti-inflammatory properties [12,13,14,15].

Plant in vitro cultures offer possibilities for the production of valuable secondary metabolites in the biomass from protected and rare plants, regardless of climatic conditions [16,17]. In order to increase the efficiency of cultures, to produce more biomass and to increase the productivity of biomass for the production of secondary metabolites, in vitro plant cultures have been adapted to be grown in special bioreactors, which can be used on an industrial scale. The following types of bioreactors can be distinguished: liquid-phase bioreactors, gas-phase bioreactors, hybrid bioreactors, and the temporary immersion system (TIS) [18]. TIS bioreactors are characterized by submerged and nonsubmerged cycles, and good gas exchange [19]. The production of plant secondary metabolites has been optimized for some cultures grown in vitro in bioreactors, e.g., camptothecin in *Camptotheca acuminata* [20]; xanthone, benzophenone, and bioflavonoids in *Cyclopia genistoides* [21].

The production of secondary metabolites in plant in vitro cultures can be stimulated by different plant growth regulators (PGRs), different basal compositions of the growth medium, and by feeding with elicitors and precursors. In experiments, precursor feeding is used to increase the production of valuable secondary metabolites. Phenylalanine (Phe) is a substrate in the first reaction of the phenylpropanoid pathway, and, for this reason, is frequently used as a precursor to stimulate the production of flavonoids, phenolic acids, lignans, and coumarins [17]. GSLs share the following common basic structural features: a *β*-thioglucose moiety, an oxime sulfonate moiety, and a variable side chain derived from aliphatic, aromatic, or indole amino acids. In general, there are the following three different types of GSLs depending on the amino acid origin: aliphatic GSLs derived from leucine, valine, methionine (Met), isoleucine, or alanine; indole GSLs derived from tryptophan (Trp); and aromatic GSLs derived from Phe or tyrosine. Phe can be used as a strategy to enhance the production of Phe-derived aromatic GSLs such as gluconasturtiin and glucotropaeolin [22]. On the other hand, Trp can be used to obtain Trp-derived indole GSLs such as glucobrassicin, 4-hydroxyglucobrassicin, and 4-methoxyglucobrassicin [23].

The aim of the present research was to evaluate the influence of precursor feeding (with Phe and Trp) on the production of GSLs, polyphenols, saccharides, and photosynthetic pigments, and also to assess the antioxidant potential and antimicrobial properties of extracts from *N. officinale* microshoot cultures grown in Plantform bioreactors.

## 2. Results and Discussion

### 2.1. Preliminary Results Obtained in Agitated Cultures

The first screening analysis had been carried out with *N. officinale* agitated microshoot cultures (Figure S1 in Supplementary Materials). All the data produced by that experiment are presented in the Supplementary Materials section.

In that experiment, we analyzed the influence of Phe and Trp additions at the beginning (day 0) and after 10 days of the growth period on the total GSL content, total and individual concentrations of polyphenol compounds, and on antioxidant activity. No negative impact of the applied precursor concentrations on the growth of biomass (Gi) was observed in any of the experimental treatments (Figure S1, Table S1). The study showed that Phe and Trp had a stimulating effect on the production of GSLs (Table S2). The study also confirmed positive effects on the concentrations of *p*-coumaric acid, ferulic acid, and rutoside in comparison with the control microshoot cultures (Table S3). The antioxidant potential was estimated using the CUPRAC method. The analysis showed an antioxidant potential of the tested microshoot cultures to be similar to that of the control cultures (Table S4).

Those results had helped us to scale up the experiments and adapt the chosen conditions to the study on *N. officinale* microshoots grown in the Plantform TIS.

### 2.2. The Appearance of Microshoot Cultures and Growth Rate

**Figure 1:**
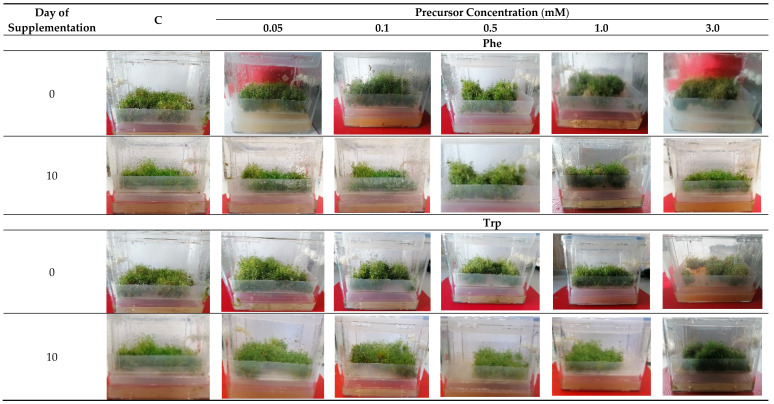
Morphological appearance of Plantform bioreactor-grown *N. officinale* microshoot cultures after precursor feeding.

For the bioreactor-grown *N. officinale* microshoot cultures, at all concentrations of Phe and Trp, a green color and large amounts of microshoots were characteristic features. The cultures grew in clumps. At the concentration of 3.0 mM Phe and Trp, a lighter green color of microshoots was observed. No negative effects on the growth of microshoot cultures were observed at any of the tested concentrations (Figure 1).

The Gi for the Phe variants ranged from 6.92 to 11.88. The highest Gi was obtained with 0.1 mM Phe (day 0) and the lowest with 0.5 mM Phe (day 10). The Gi for the Trp variants ranged from 5.47 to 13.66. The highest Gi was obtained with 3.0 mM Trp (day 0) and the lowest with 3.0 mM Trp (day 10). For the control cultures (C), the Gi was 12.23 for day 0, and 8.32 for the microshoots that had been supplemented with H_2_O on day 10 of the growth period (Table 1).

Adding the precursors at the beginning (day 0) caused a slight decrease in biomass growth. In contrast, the addition of the precursors on day 10 resulted in the following greater biomass increments: Gi = 11.48 for 3.0 mM Phe and Gi = 13.66 for 3.0 mM Trp, which were, respectively 1.4 and 1.6 times more than the corresponding Gi values for C. In our previous study [24], *N. officinale* microshoots had been maintained in other TIS bioreactors, namely RITA^®^ (Vitropic, France). The highest Gi (31.71) was obtained after 20 days of growth, which was 2.3 times higher than the highest results obtained with 3.0 mM Trp (Table 1). Additionally, a study by Weremczuk-Jeżyna et al. [25] demonstrated better growth of *Dracocephalum forrestii* transformed shoots grown in a RITA^®^ bioreactor in comparison with a Plantform bioreactor. The authors suggested that that effect may have resulted from the lower availability of oxygen to the shoots while being flooded in the Plantform bioreactor. Our *N. officinale* microshoot cultures grown in the Plantform bioreactor were characterized by higher growth in comparison with the agitated cultures studied by us earlier in order to optimize the basal media composition (Figure S1, Table S1). In fact, the maximum Gi obtained (13.66) was 1.3 times higher than the best results reached with the agitated cultures grown on MS medium containing 1 mg/L zeatin (Zea) and 1 mg/L NAA [26] (Table 1). The Gi values for *N. officinale* grown in Plantform bioreactors are relatively high in comparison with the 30-day microshoot cultures of *S. chinensis* cv. Sadova No.1, for which the values of Gi were about three times lower [27].

### 2.3. Phytochemical Analyses of Metabolites

#### 2.3.1. Total Soluble Saccharide Content

The total amount of soluble saccharides (TWSC) was dependent on the precursors added, their concentrations, and also the time of supplementation. In the case of Phe, the total amount of soluble saccharides ranged from 4.34 to 6.84 g glucose (GLU) equivalent/100 g dry weight (DW). The highest soluble saccharide content was obtained with 0.1 mM Phe (day 0), and the lowest with 3.0 mM Phe (day 0). For C cultures, the soluble saccharide content was 4.65 g GLU/100 g DW (day 0) and 5.57 g GLU/100 g DW (day 10) (Table 2).

In comparison with C, the total amount of soluble saccharides increased after the addition of Phe at the concentrations of 0.1, 0.5, and 1.0 mM Phe (day 0), and 0.1 and 0.5 mM Phe (day 10). With Trp added on day 0, only the concentration of 3.0 mM caused a decrease in the total amount of soluble saccharides. However, the 3.0 mM Trp added on day 10 increased the amount of soluble saccharides. The highest amount of saccharides was obtained with 0.05 mM Trp (day 0), which was 1.2 times higher than in C (Table 2). To the best of our knowledge, there have been no studies on the stimulation of the production of soluble saccharides in plant in vitro cultures treated with Phe or Trp as precursors.

#### 2.3.2. Photosynthetic Pigment Content

The evaluation proved a generally positive influence of the precursors on the concentration of photosynthetic pigments in *N. officinale* microshoot biomass.

The chlorophyll *a* content ranged from 35.49 to 140.18 mg/100 g DW. In C, the highest value for chlorophyll *a* (64.36 mg/100 g DW) was obtained with supplementation on day 0. The lowest value was obtained with 0.1 mM Trp (day 10). The maximum content was obtained for microshoots grown with 1.0 mM Trp (day 0), which was 2.2 times higher than in C (Table 3).

The chlorophyll *b* content in extracts from the experimental *N. officinale* cultures varied from 29.15 to 148.24 mg/100 g DW. In C, the highest value (77.03 mg/100 g DW) for chlorophyll *b* was obtained for day 0 of the growth period. The lowest content was obtained with 0.05 mM Phe (day 10). The maximum value was obtained with 3.0 mM Trp (day 0), which was 1.9 times higher than in C (Table 3).

The total amount of chlorophylls *a* + *b* in the analyzed extracts ranged from 64.70 to 236.39 mg/100 g DW. The lowest content was obtained with 0.05 mM Phe (day 10). The maximum value was obtained with 3.0 mM Trp (day 0). The maximum total amount of chlorophylls *a* + *b* (141.39 mg/100 g DW) in C was determined for microshoots supplemented on day 0 (Table 3).

The amount of carotenoids in extracts of the experimental *N. officinale* cultures varied from 0.00 to 21.72 mg/100 g DW. The lack of carotenoids occurred with 0.05 mM Trp (day 10) and 3.0 mM Trp (day 0). The maximum total amount of carotenoids (7.37 mg/100 g DW) in C was confirmed for the microshoots supplemented on day 10 of the growth period. The highest amount of carotenoids was obtained with 0.5 mM Phe (day 0), which was 2.9 times higher than in C (Table 3).

To the best of our knowledge, there have been no studies on the stimulation of the production of chlorophylls or carotenoids in plant in vitro cultures treated with Phe or Trp as precursors.

#### 2.3.3. Total GSL Content

The total amount of GSLs, determined using the spectrophotometric assay, was dependent on the precursors added, their concentrations, and also the time of supplementation (Table 4). In general, the total amounts of GSLs in the samples treated with Phe or Trp, respectively, were higher when compared to the control (C). An important factor was the day of precursor addition. The highest results were obtained with the precursors added at the beginning of culture growth. However, the best time of supplementation with precursors may depend on the tested plant species grown in vitro, e.g., in a *Cistanche deserticola* culture, the best results of phenylethanoid glycoside production had been obtained after supplementation with Phe on day 8 of culture growth [28]. In other experiments, e.g., with *Citrullus colocynthis* [29] and *Abutilon indicum* [30], in which Phe was also tested, the results were best when the culture media were supplemented with Phe on day 0.

#### 2.3.4. UHPLC-DAD-MS/MS Analysis of GSLs

The samples with the highest estimated amounts of GSLs for each precursor (3.0 mM Phe, 3.0 mM Trp, added on day 0) obtained by spectrophotometric screening were subjected to qualitative and quantitative analyses using UHPLC-DAD-MS/MS. The following five GSLs were found in the control sample: Met-derived 7-(methylsulfinyl)heptyl GSL (**1**), glucohirsutin (**2**), Phe-derived gluconasturtiin (**3**), Trp-derived glucobrassicin (**5**), and 4-methoxyglucobrassicin (**6**). Additionally, extracts from the microshoots supplemented with 3.0 mM Phe contained Trp-derived GSL 4-hydroxyglucobrassicin (**4**). In the microshoots supplemented with 3.0 mM Trp, the following four GSLs were detected: Phe-derived **3** and Trp-derived **4**, **5**, and **6** (Table 5, Figure S2).

The amounts of individual GSLs ranged from 3.78 to 149.99 mg/100 g DW. The main GSLs were **6**, **3**, and **5**. For **6**, the amount ranged from 76.12 to 149.99 mg/100 g DW with 3 mM Trp and 3.0 mM Phe, respectively. When compared to the control microshoots, the amount of **6** increased significantly, i.e., 6.0 and 11.7 times, respectively. The amount of **3** decreased with 3 mM Trp and increased with 3.0 mM Phe, when compared to the control microshoots (15.65 mg/100 g DW). For **5**, the amount ranged from 3.78 to 6.78 mg/100 g DW, which represented an increase when compared to the control (traces).

The productivity for the main compounds was also calculated. The maximum concentrations of **6**, **3**, and **5** were obtained with 3.0 mM Phe, and the productivity for these compounds was 398.77, 97.59, and 18.03, respectively. The calculated productivity for **6** was 13.6 times higher with 3.0 mM Phe than in the control. For **3**, the highest productivity was obtained with 3.0 mM Phe, which was 2.7 times higher than in the control. The productivity for **3** with 3.0 mM Trp was 2.7 times lower than in C (Table 5, Figure S2).

In our previous study [26], the analysis of the total GSL content had been performed in extracts from *N. officinale* microshoots grown in agar and agitated cultures on the same MS medium containing 1 mg/L BA and 1 mg/L NAA. The highest total amount of GSLs (182.80 mg/100 g DW) was then obtained in agitated cultures after 10 days of growth, which was 1.5 and 1.1 times lower in comparison, with the best results obtained with 3.0 mM Phe and 1.0 mM Trp (Table 5).

An UHPLC-DAD-MS/MS analysis of GLSs in *N. officinale* microshoot cultures grown in a RITA^®^ bioreactor had also been performed [24]. Taking into account our previous and present results, the differences in the production of GSLs between the bioreactors used were evident. In the RITA^®^ bioreactor, the microshoots grown on MS medium with 1 mg/L BA and 1 mg/L NAA had produced a 3.6 times higher amount of **3** after 20 days of growth in comparison with the control without precursors. The highest amount of **3** in extracts from the *N. officinale* microshoot cultures after feeding with 3.0 mM Phe was 1.5 times lower than in *N. officinale* grown in the RITA^®^ bioreactor. For **6**, after feeding with the precursors, a 3.0- and 1.5-times higher content, with 3.0 mM Phe and 3.0 mM Trp, respectively, was obtained than in the microshoots grown for 20 days in the RITA^®^ bioreactor. The amount of **5** with 3 mM Phe and Trp was 1.1 and 2.0 times lower, respectively, than in the *N. officinale* microshoot cultures grown for 20 days in the RITA^®^ bioreactor (Table 5, Figure S2).

Wielanek et al. [31] studied the amounts of **3** in *N. officinale* hairy root cultures grown with precursors (Phe, Cysteine (Cys), methionine (Met), serine (Ser)) at a concentration of 0.5 mM added to 12-day-old cultures and collected 10 days after feeding. The study found that Phe stimulated the production of **3** more than of other amino acids. In the same study, stimulation of the production of **3** was observed in *Barbarea verna* hairy root cultures when supplemented with 0.5 mM Phe and 0.5 mM Cys [31].

#### 2.3.5. Total Flavonoid Content

The production of flavonoids in the studied microshoot cultures grown in Plantform bioreactors was dependent on the kind of precursor, its concentration, and the time of supplementation. The total amount of flavonoids obtained with Phe ranged from 808.38 to 1364.38 mg rutoside equivalent (RE)/100 g DW. For the C cultures, the flavonoid content was 565.16 (day 0) and 863.71 mg RE/100 g DW for microshoots supplied with H_2_O (day 10). The lowest amount was obtained with 0.5 mM Phe (day 10). The best results were obtained with 3.0 mM Phe and 1.0 mM Trp (day 0), which were 1364.38 and 1324.14 mg RE/100 g DW, respectively (Table 4). They were 2.4 and 2.2 times higher than in the C microshoots (Table 4).

In our previous study [24] with *N. officinale* microshoot cultures grown in RITA^®^ bioreactors, the total amount of flavonoids had also been determined. The highest total flavonoid content obtained in the present study with 3.0 mM Phe (day 0) was 2.4 times higher than in the *N. officinale* microshoot cultures grown in the RITA^®^ bioreactors for 20 days (Table 4). A study by El-Hawary et al. [32] confirmed the influence of Phe on the total flavonoid content in *Sequoia sempervirens* callus cultures. In that study, the concentration of 200 mg/L Phe caused a two-fold increase in the amount of flavonoids in comparison with C. The same effect was obtained in our study with 3 mM Phe (day 0). The feeding with 0.05 mM and 0.5 mM Phe had not changed the total amount of flavonoids in *Ocimum basilicum* cv. Grand Vert in vitro cultures. In our study, the same concentrations of Phe increased the production of flavonoids in *N. officinale* microshoot cultures [33].

#### 2.3.6. Total Polyphenol Content

The influence of the applied precursor feeding on polyphenol production determined using the F-C method was noticeable. The total amount of polyphenols obtained with Phe ranged from 228.29 to 325.55 mg gallic acid equivalent (GAL)/100 g DW. The highest total polyphenol content (325.55 mg GAL/100 g DW) was obtained with a Phe concentration of 0.1 mM (day 0), which was 1.7 times higher than in C. Phe had a smaller influence on the amount of polyphenols compared to Trp. The total amount of polyphenols obtained with Trp varied from 151.43 to 1062.76 mg GAL/100 g DW. The highest polyphenol content was obtained with 3.0 mM Trp added on day 0 and day 10 of the growth period, which was 4.6 and 4.3 times higher than in C, respectively. For the C cultures, the polyphenol content was 189.61 mg GAL/100 g DW for day 0 and 248.02 mg GAL/100 g DW for the microshoots supplied with H_2_O on day 10 of the growth period (Table 4).

Our previous study [24] also determined the total amount of polyphenols for the *N.officinale* microshoot cultures grown in RITA^®^ bioreactors. The amounts of polyphenols obtained in the present study were higher than those in the *N. officinale* microshoot cultures grown in the RITA^®^ bioreactors for 20 days. The highest total polyphenol content was obtained with 3.0 mM Trp (day 10), which was 2.0 times higher than in *N. officinale* microshoot cultures grown in the RITA^®^ bioreactors for 20 days (Table 4). El-Hawary et al. [32] also determined the polyphenol content in *S. sempervirens* callus cultures. In their study, they obtained about a two-fold increase in polyphenol content in cultures grown in vitro with Phe. Another study, by Koca and Karaman [33], also found a stimulating effect of 0.05 mM and 0.5 mM Phe on the total production of polyphenols in *O. basilicum* cultures in vitro. In our study, with these concentrations of Phe added on day 0, we too observed an increase in the total amount of these compounds.

#### 2.3.7. HPLC-DAD Analysis of Polyphenol Compounds

Using the HPLC-DAD method, two phenolic acids (*p*-coumaric and ferulic) and one flavonoid (rutoside) were estimated in extracts of the experimental *N. officinale* microshoot cultures grown in Plantform bioreactors. The quantitative analysis confirmed the stimulatory effect of the precursors on the amounts of individual compounds estimated. The highest *p*-coumaric acid content was obtained with 0.5 mM Trp (day 10) (29.11 mg/100 g DW), which was 2.6 times higher than in C. For ferulic acid, the best result was obtained with 3.0 mM Phe (day 0) (27.76 mg/100 g DW), which was 7.6 times higher than in C. For rutoside, the highest amount was obtained with 0.1 mM Trp (day 10) (16.03 mg/100 g DW), which was 4.2 times higher than in C (Table 6).

There have been no studies on the stimulation of the production of polyphenol compounds in plant in vitro cultures by the addition of Trp to the growth medium. There are known studies in which feeding plant in vitro cultures with Phe increased the production of flavonoids and phenolic acids. Phe is the substrate in the first reaction of the phenylpropanoid pathway. As a result of the phenylpropanoid pathway, secondary metabolites such as flavonoids, phenolic acids, coumarins, and lignans are formed [17]. Feeding with Phe has been found to increase the production of flavonoids (cinaroside, rutoside, casticin) and phenolic acids (neochlorogenic, *p*-hydroxybenzoic, caffeic and *p*-coumaric acid) in *Vitex agnus castus* L. microshoot cultures [34]. Another study, by Szopa et al. [35], also confirmed a stimulatory effect on the production of phenolic acids in *Aronia arbutifolia* and *Aronia melanocarpa* shoot cultures. In that study, higher amounts had been obtained when 0.1 mM Phe (*A. melanocarpa*) and 5 mM Phe (*A. arbutifolia*) was added on day 10 of the growth periods. In our *N. officinale* microshoot extracts, higher amounts were obtained when 3.0 mM Phe was added on day 0 (Table 4 and Table 6). An increased production of flavonoids such as quercetin has been obtained in callus cultures of *Citrullus colocynthis* [29] and *Abutilon indicum* [30].

### 2.4. Antioxidant Activity

The following three assays: CUPRAC, DPPH, and FRAP, were used to measure the antioxidant potential of *N. officinale* microshoot extracts treated with precursors.

For the CUPRAC and FRAP methods, the maximum antioxidant activity was obtained with 0.1 mM Phe (day 0), respectively 3.05 and 0.94 mmol trolox equivalent (TE)/100 g DW, which was 2.0 and 3.9 times higher than for the corresponding estimations for C (2.15 and 0.40 mmol TE/100 g DW, respectively) (Table 7). For the DPPH assay, the maximum antioxidant activity was obtained with 0.5 mM Trp (day 10) (0.90 mmol TE/100 g DW), which was 1.3 times higher than in C (0.69 mmol TE/100 g DW) (Table 7).

In general, the strongest antioxidant potential of *N. officinale* microshoot extracts estimated with all the methods used (CUPRAC, DPPH, and FRAP) was obtained after the addition of 3.0 mM Phe on day 0. The antioxidant power for this strategy of precursor feeding was, respectively 2.0, 1.8, and 3.0 times higher than the corresponding values for C. High amounts of flavonoids, individual polyphenol compounds, and carotenoids were obtained on this particular experimental variant. This suggests the potential influence of these metabolites on antioxidant power (Table 4, Table 6 and Table 7).

### 2.5. Principal Component Analysis (PCA)

**Figure 2:**
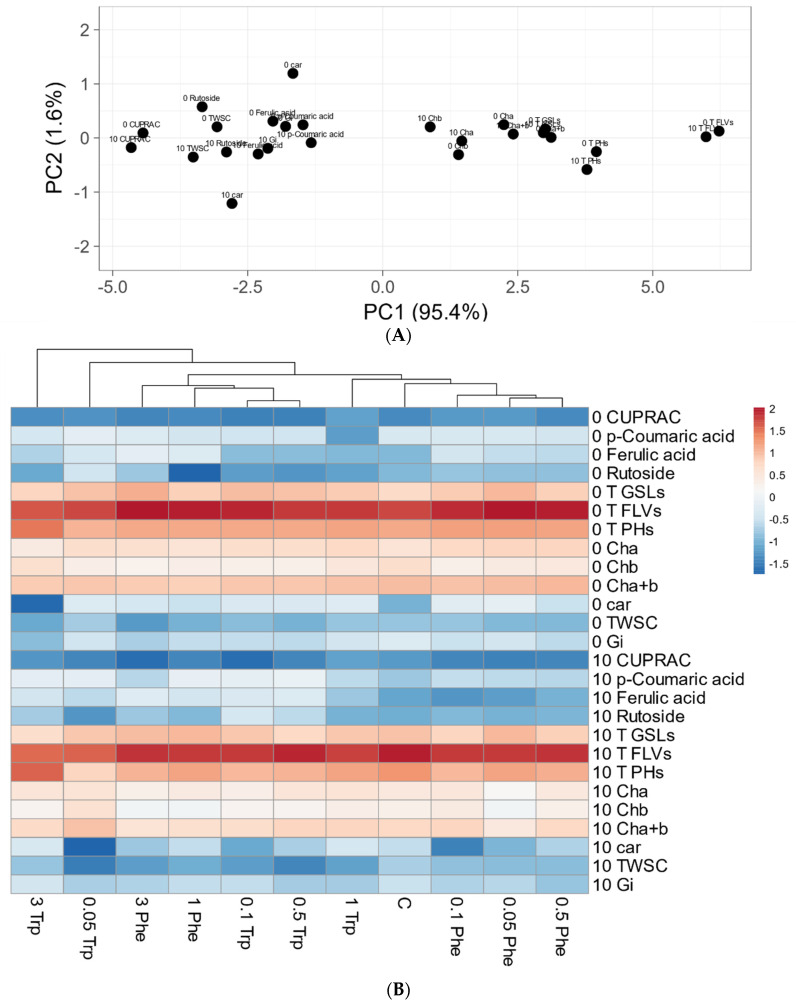
Principal component analysis (PCA) plot of measured bio-chemical parameters for plant material (panel **A**) and heat map of biochemical parameters of different treatments (panel **B**). Original values are ln(x)-transformed. Unit variance scaling is applied to rows; singular value decomposition (SVD) with imputation is used to calculate principal components. X and Y axis show principal component 1 (PC1) and principal component 2 (PC2) that explain 95.4 and 1.6% of the total variance, respectively. In heat map columns are centered; unit variance scaling is applied to columns. Columns are clustered using correlation distance and average linkage [36].

PCA plot presents two principal components PC1 and PC2 that explain 95.4 and 1.6% of the total variance, respectively (Figure 2). The first principal component increases with parameters concerning secondary metabolism as total pool of flavonoid, phenolics, glucosinolates, and photosynthesis-related chlorophylls accumulation. The strongest positive correlation is observed with the flavonoid pool. A negative correlation occurs for selected phenolic acids, and pools of soluble carbohydrates and carotenoids. The second PC correlates with the carotenoid pool in both vegetation periods, culture day 0 positively and after 10 days negatively. The presented heat map shows these data matrix and coloring gives an overview of the numeric differences. The high values of supplemented precursors cluster together, whereas the plants supplemented with low concentrations of Phe and Trp clusters around the control plants (Figure 2).

### 2.6. Antimicrobial Activity

**Figure 3:**
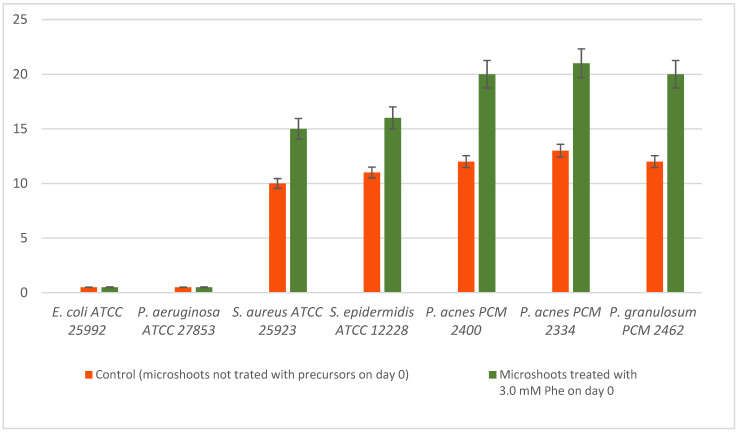
Zones of bacterial growth inhibition by the tested *N. officinale* extracts (mm).

The initial diffusion test was aimed at assessing the antibacterial properties of the microshoot *N. officinale* extracts (microshoot culture grown with 3.0 mM Phe (day 0) and microshoots not treated with the precursors on day 0 as non-modified control). The presence of zones of bacterial growth inhibition around the applied samples indicated their antibacterial activity. The data contained in Figure 3 clearly show that of the two tested samples, the most active was the extract from *N. officinale* microshoot cultures grown with 3.0 mM Phe (day 0). This extract most strongly inhibited the growth of all the Gram-positive microaerobic *Propionibacterium* spp. (20–21 mm), and less strongly of the Gram-positive aerobic strains (15–16 mm). The antimicrobial activity of this extract was most likely connected with the estimated amounts of biologically active metabolites. Weaker activity was shown by the extract from the C culture, which created inhibition zones against the microaerobic and aerobic acne strains in the range of 13–12 mm and 11–10 mm, respectively.

Both extracts had a narrow spectrum of activity directed against Gram-positive strains. Neither of them were active against Gram-negative bacteria.

Next, the minimum inhibitory concentration (MIC) value was determined for the two kinds of *N. officinale* extracts from microshoot cultures (grown with 3.0 mM Phe added on day 0, or from C—control microshoots not treated with precursors on day 0), which in the above microbiological test showed activity against some strains. A series of microdilutions was performed in 96-well plates with the appropriate bacterial media. The experiment included positive, negative, and reagent dye controls. The wells with the medium and plant solutions were inoculated with aerobic (*S. aureus* or *S. epidermidis*) and microaerobic (*P. acnes* PCM 2400, *P. acnes* PCM 2334 or *P. granulosum*) bacteria. After incubation, the plates were read in a plate reader.

The MIC data in Table 8 confirmed the results obtained in the agar screening test (Figure 3). The broadest spectrum of activity against all the tested Gram-positive microaerobic bacteria and significant activity against these acne bacteria was shown by the Phe-modified *N. officinale* microshoot extract, with the MIC value in the range of 250–500 µg/mL. The same extract reached an MIC of 1000 µg/mL against Gram-positive aerobic strains. The *N. officinale* control herb extract achieved significantly weaker MICs (500–4000 µg/mL) compared to the *N. officinale* microshoot cultures.

Subsequently, the minimum bactericidal concentration (MBC) can be determined from the MIC test by sub-culturing on agar plates. Thus, based on MIC plates, the clear medium was spread on agar and incubated to confirm its sterility. The concentration of the sample that did not produce colonies was considered as the MBC value. Using the MBC/MIC ratio, we determined the type of antibacterial action. If the ratio MBC/MIC was ≤4, the effect was considered as bactericidal, but if the ratio MBC/MIC was >4, the effect was defined as bacteriostatic [37]. The MBC/MIC ratio is displayed in Table 8. None of the plant extracts showed any bactericidal activity against the tested bacteria. The most promising activity was displayed against the microaerobic Gram-positive acne strains by the Phe-modified extract from the *N. officinale* microshoots. The C extract showed only a slight bacteriostatic effect, or not a measurable one. Probably, isothiocyanates (products of enzymatic decomposition of GSLs), which are the subject of the latest research on this biological activity of these extracts, may be responsible for the antimicrobial activity of the compounds [38].

### 2.7. Cytotoxicity towards Normal Human Fibroblast Cells

**Figure 4:**
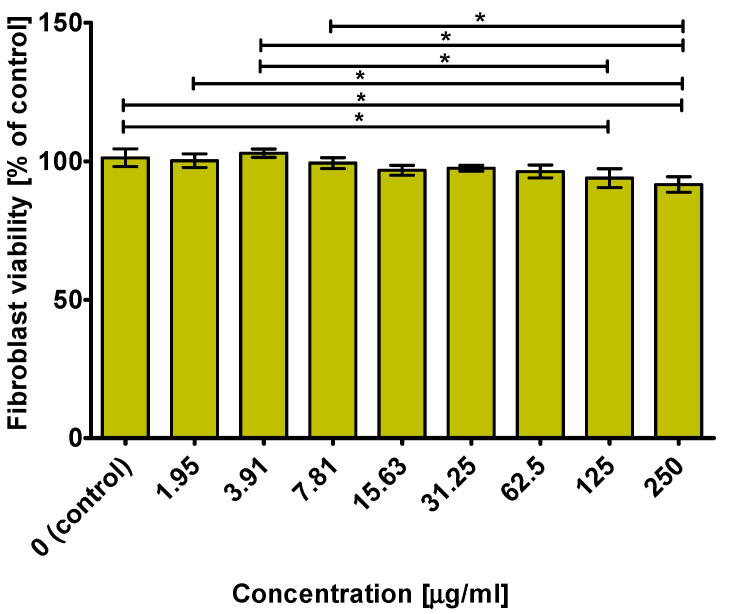
Fibroblast viability after 48 h incubation with extract from *N. officinale* grown with 3.0 mM Phe (day 0). The results were obtained using MTT assay. * Significantly different data between tested groups. *p* < 0.05; one-way ANOVA followed by Tukey’s multiple comparison test.

After a 48-h incubation, it was demonstrated that the extract from the *N. officinale* microshoot cultures grown with 3.0 mM Phe (day 0) at concentrations of 1.95–62.5 μg/mL did not significantly inhibit fibroblast viability compared to the control (culture medium, 0 μg/mL). However, a slight decrease in cell viability to approximately 93 and 92% was observed at the highest tested concentrations (125 and 250 μg/mL) (Figure 4). This result means that the CC_50_ value of the *N. officinale* microshoot extract was higher than 250 μg/mL.

## 3. Materials and Methods

### 3.1. Experimental In Vitro Cultures

Initial microshoot cultures of *N. officinale* were established and maintained as reported previously [39]. That study had involved the cultivation of *N. officinale* microshoots in the Plantform^TM^ (Plant form, Hjärup, Sweden) temporary immersion system (TIS) containing 500 mL of the Murashige and Skoog (MS) medium [40] with 3% (*w*/*v*) sucrose and supplemented with 1 mg/L 6-benzyladenine (BA) and 1 mg/L 1-naphthaleneacetic acid (NAA). The medium composition had been identified in our previous study as being optimal for the cultivation of microshoots [26]. The inoculum used in this study was composed of 10 g of FW (fresh weight) of microshoots. The microshoots were grown under continuous exposure to LED white light (2.75 W/m^2^) at a temperature of 25 ± 2 °C. The immersion cycle was set to 5 min every 1.5 h, at an aeration rate of 1.0 vvm.

### 3.2. Procedure for Precursor Feeding

Sterile stock solutions of precursors were added to the experimental cultures at the beginning of the growth period (day 0) or on day 10 of the growth period. The precursors used were Phe and Trp. The growth medium was fed the following concentrations of these precursors: 0.05, 0.1, 0.5, 1.0, and 3.0 mM. The experimental media and biomass samples were collected after 20 days of the growth period (3 series, *n* = 6).

Stock solutions of Phe (L-phenylalanine, Sigma-Aldrich, St. Louis, MO, USA) were prepared by dissolving 20.85 (0.05 mM), 41.73 (0.1 mM), 208.55 (0.5 mM), 417.10 (1.0 mM), and 1251.30 (3.0 mM) mg Phe in 25 mL of distilled H_2_O. These solutions were sterilized using a 0.22-micrometer syringe filter (Millex^®^GP; Merck Millipore, Burlington, MA, USA), and 5 mL of each stock solution was added to the culture medium to give the desired concentration in the medium.

Stock solutions of Trp (L-tryptophan, Sigma-Aldrich, St. Louis, MO, USA) were prepared by dissolving 25.75 (0.05 mM), 51.50 (0.1 mM), 257.50 (0.5 mM), 515.00 (1.0 mM), and 1545.00 (3.0 mM) mg Trp in 25 mL of distilled H_2_O. These solutions were sterilized using a 0.22-micrometer syringe filter (Millex^®^GP; Merck Millipore, Burlington, MA, USA), and 5 mL of each was added to the culture medium to give the desired concentration in the medium.

Microshoots were also cultivated as control cultures (C) by being grown without the precursors. At the beginning (day 0) or on day 10 of the growth period, redistilled sterile H_2_O was added to the bioreactor in a volume corresponding to that used with the precursors (5 mL).

### 3.3. Calculating the Growth Index

Biomass increments were calculated using the growth index (Gi). The biomass of microshoots collected after 20 days was dried and lyophilized (Labconco Corporation, Kansas City, MO, USA), and weighed (DW—dry weight). The Gi was calculated using the following formula: Gi = $\frac{\left({\mathrm{Dw}}_{1}-{\mathrm{Dw}}_{0}\right)}{{\mathrm{Dw}}_{0}}$, where Dw_1_—dry weight of the microshoots obtained at the end of growth periods and Dw_0_—dry weight of the inoculum [41].

### 3.4. Biomass Extraction

The biomass harvested from the tested *N. officinale* microshoot cultures was immediately frozen in liquid N_2_ and lyophilized (Labconco Corporation, Kansas City, MO, USA). The biomass was pulverized in a mixing ball mill (MM400, Retch, Haan, Germany). Samples (0.2 g) were weighed out and extracted twice in 4 mL of methanol (STANLAB, Lublin, Poland) under sonication for 20 min in an ultrasonic bath (POLSONIC 2, Warsaw, Poland). Then, the samples were centrifuged (8 min, 2000× *g*; MPW-223E; MPW, Warsaw, Poland) and filtered (0.22 μm syringe filters; Millex^®^GP; Merck Millipore, Burlington, MA, USA). If not otherwise stated, the extract was used for further analyses.

### 3.5. Phytochemical Analyses of Metabolites

#### 3.5.1. Determination of Total Soluble Saccharides

A modified Dubois et al. [42] phenol-sulphuric method was used for the analysis of soluble saccharides [43]. Briefly, samples were extracted in H_2_O (5 mg per 1.5 mL). The extract was diluted with water to fit the linearity range of the method, then an equal volume of 5% phenol solution was added. After mixing, concentrated sulphuric acid was added and the samples were incubated for 20 min, and then transferred to 96-well plates. The absorbance at 490 nm was measured (Synergy II, Biotek, Winooski, VT, USA). The saccharide content was expressed as the GLU equivalent.

#### 3.5.2. Analysis of Photosynthetic Pigments

Chlorophylls and carotenoids were estimated spectrophotometrically according to Czyczyło-Mysza et al. [44]. Plant material was extracted in 96% ethanol, centrifuged, and transferred to 96-well micro-plates, and the absorbance was read at 470, 648, and 664 nm (Synergy II). The concentrations of chlorophyll *a*, chlorophyll *b*, total chlorophyll (*a* + *b*), and total carotenoids (c) were calculated with Lichtenthaler and Buschman (2001) equations.

#### 3.5.3. Spectrophotometric Analysis of the Total GSL Pool

The analysis of GSLs was performed with the method of Gallaher et al. [45], as was described in our previous studies [24,26,39], utilizing the ferricyanide reaction. Briefly, samples were extracted under inactivated myrosinase conditions. The supernatants were evaporated, and the residue was re-dissolved in H_2_O and cleaned employing anion exchange SPE (Supel-Select SAX, 60 mg, 3 mL, Bellefonte, PA, USA). The purified GSLs were hydrolyzed with 1 M NaOH, and after 30 min the samples were neutralized with concentrated HCl. Standard or sample were mixed with the ferricyanide solution in a 96-well plate format. Absorbance was read at 420 nm (Synergy II, BioTek, Winooski, VT, USA). Sinigrin was used as a calibration standard. The results of the total GSL content are expressed as mg of sinigrin (SIN)/100 g dry weight (DW). All technical details are given by Klimek-Szczykutowicz et al. (2019, 2020b, a).

#### 3.5.4. Total Flavonoid Assay

Total flavonoid content was estimated spectrophotometrically according to Ramos et al. [46]. A 100-microliter aliquot of methanolic extract was mixed with 40 μL of 10% AlCl_3_ in a total of 1 mL made up with 5% acetic acid. After 20 min, the samples were transferred to 96-well plates. The absorbance was measured at 425 nm (Synergy II). The amounts of flavonoids were expressed as mg of RE/100 g DW. The analysis was performed in triplicate (including reagent blanks).

#### 3.5.5. Total Phenolic Assay

Estimation of total phenolic content was performed according to the Singleton method [47] with modifications [43]. The Folin-Ciocalteu (F-C) phenol reagent was mixed with the analyzed extracts (100 μL). After 10 min, the same volume of saturated Na_2_CO_3_ (0.45 mL) was added. The samples were incubated in the dark for 2 h, and, after centrifugation, transferred to 96-well plates. The absorbance was detected at 760 nm (Synergy II). The pool of phenolic compounds was expressed as mg of GAL/100 g DW. The analysis was performed in triplicate (including reagent blanks).

#### 3.5.6. Analysis of GSL Content with UHPLC-DAD-MS/MS

Isolation of desulfoglucosinolates (dGSLs) was performed as reported previously [48,49] from 100 mg of dried plant material. The plant material was firstly subjected to extraction in MetOH/H_2_O (70:30 *v*/*v*; Gram-Mol d.o.o., Zagreb, Croatia). The supernatant was loaded on mini-columns filled with DEAE-Sephadex A-25 anion-exchange resin (Sigma-Aldrich, St. Louis, MO, USA) and the columns were then washed to remove the remaining non-polar compounds. To create optimal conditions for the sulfatase reaction, the mini columns were washed with 20 mM NaOAc buffer (Merck, Darmstadt, Germany), followed by the addition of sulfatase (type H-1 from *Helix pomatia*; Sigma-Aldrich, St. Louis, MO, USA). The reaction was left overnight and the dGSLs were eluted the next day with ultrapure H_2_O (Merck Millipore, Burlington, MA, USA). The standard used—sinigrin—was obtained from Sigma Aldrich; glucohirsutin (**2**), gluconasturtiin (**3**), 4-hydroxyglucobrassicin (**4**), glucobrassicin (**5**), and 4-methoxyglucobrassicin (**6**) were obtained from Phytoplan (Heidelberg, Germany). All other chemicals and reagents were of analytical grade.

Analysis was performed using the UHPLC-DAD-MS/MS method (Ultimate 3000RS with TSQ Quantis MS/MS detector, Thermo Fischer Scientific, Waltham, MA, USA) using a Hypersil GOLD column (3.0 µm, 3.0 × 100 mm, Thermo Fischer Scientific, USA). A gradient consisting of solvent A (50 μM NaCl in H_2_O) and solvent B (acetonitrile:H_2_O 30:70 *v*/*v*) was applied at a flow rate of 0.5 mL/min as follows: 0.14 min, 96% A and 4% B; 7.84 min, 14% A and 86% B; 8.96 min, 14% A and 86% B; 9.52 min, 5% A and 95% B; 13.16 min, 5% A and 95% B; 13.44 min, 96% A and 4% B; 15.68 min, 96% A and 4% B. The column temperature was held at 15 °C, and the injection volume was 2 µL. The system was operated in the positive ion electrospray mode and the electrospray interface was H-ESI operating with a capillary voltage of 3.5 kV at 350 °C. The signals were recorded at 227 nm using a DAD detector. Quantification of dGSLs was performed using an external calibration curve of pure desulfosinigrin (range from 13.56–542.50 µM). For each individual dGSL, a response factor (RPF) was taken in accordance with the literature, as follows: RPF 1.1 for 2 [50], 0.95 for 3, 0.28 for 4, 0.29 for 5, 0.25 for 6 [51]; arbitrary 1.0 for 7-(methylsulfinyl)heptyl GSL (**1**) (Figure S2).

#### 3.5.7. Analysis of Polyphenol Compounds Using HPLC-DAD

The analysis was performed with the HPLC-DAD method described previously [52,53]. For these estimations, the methanolic extracts were used (prepared as described in Section 3.4). An HPLC-DAD system (Merck-Hitachi, Merck KGaA, Darmstadt, Germany) and a Purospher RP-18e analytical column (4 × 250 nm, 5 mL; Merck) were used. Elution was performed with a mobile phase A (methanol:0.5% acetic acid, 1:4 *v*/*v*) and a mobile phase B (methanol). The gradient program was used. The temperature was set at 25°C, the flow rate at 1 mL/min, the injection volume at 20 μL, and the detection wavelength at 254 nm (UV spectra were recorded in the 220–350 nm range). Quantitative analyses were carried for the following compounds identified previously using the UHPLC-DAD-ESI-MS method [24]: *p*-coumaric acid, ferulic acid, and rutoside (Sigma-Aldrich Co., St. Louis, MO, USA).

### 3.6. Antioxidant Activity Assays

#### 3.6.1. CUPRAC Assay

The CUPRAC method [54] adapted to the 96-well plate format [55] was used to determine the total antioxidant activity in the extracts from the tested biomass. Equal volumes of the methanolic extracts, 10 mmol/L Cu^2+^, 7.5 mmol/L neocuproine, and 1 mol/L ammonia-acetate buffer (pH 7.0) were mixed, and the samples were incubated (15 min at 25 °C). Absorbance was measured at 425 nm (Synergy II). The antioxidant pool was expressed as mmol TE/100 g DW. The measurements were performed in triplicate (including reagent blanks).

#### 3.6.2. FRAP Assay

The FRAP method [56] was additionally used for estimating the antioxidant potential. A 150-microliter aliquot of 10 mmol/L solution of TPTZ (2,4,6-tris(2-pyridyl)-s-triazine) in 40 mmol/L HCl mixed with 20 mmol/L of FeCl_3_·6H_2_O and 300 mmol/L of pH 3.6 acetate buffer (1/1/10 *v*/*v*/*v*) was mixed with 50 μL of extract. The absorbance of the sample was read at 593 nm (Synergy II) after 5-min incubation. The measurements were performed in triplicate (including reagent blanks).

#### 3.6.3. DPPH Radical-Scavenging Activity Assay

The free radical-scavenging activity of the extracts was determined using the stable radical DPPH [57]. Plant extract (50 μL) was added to 150 μL of DPPH methanolic solution. The sample was mixed and incubated for 60 min, and then its absorbance was read at 517 nm (Synergy II). The measurements were performed in triplicate (including reagent blanks).

### 3.7. In Vitro Antimicrobial Assays

The antibacterial potency of modified culture (microshoots treated with 3.0 mM Phe on day 0) and control (microshoots not treated with precursors on day 0) was evaluated using bacterial strains causing skin diseases. We used the following microaerobic Gram-positive bacteria: *Propionibacterium acnes* PCM 2334, *Propionibacterium acnes* PCM 2400, *Propionibacterium granulosum* PCM 2462 (Polish Collection of Microorganisms PCM, Institute of Immunology and Experimental Therapy, Polish Academy of Sciences, Poland); the following aerobic Gram-positive strains: *Staphylococcus epidermidis* ATCC 12228 and *Staphylococcus aureus* ATCC 25923; and the following aerobic Gram-negative strains: *Pseudomonas aeruginosa* ATCC 27853 and *Escherichia coli* ATCC 25992. Each bacterial strain was pre-incubated overnight at 37 °C on agar plates. The Mueller–Hinton (BioMaxima S.A., Lublin, Poland) agar or broth (MH-agar, MH-broth) for aerobic strains and Brain–Heart Infusion (Oxoid Ltd., Basingstoke, England) agar or broth (BHI-agar, BHI-broth) for microaerobic bacteria were used. The bacterial growth was harvested using 5 mL of sterile 0.9% NaCl; the absorbance of this inoculum was adjusted to 10^8^ CFU/mL (0.5 McFarland scale).

The disc diffusion assay can evaluate the antibacterial activity of tested extracts [58]. About 20 mL of appropriate agar medium was poured into sterile Petri dishes and a solid medium was prepared using a cotton swab and a 0.5 McFarland inoculum. The plant extracts were dissolved in DMSO (40 mg/mL), then loaded—on agar—over sterile filter paper discs (6 mm in diameter) to obtain the final concentration of 0.4 mg per disc. The obtained plates were incubated at 37 °C for 24 h (aerobic stains) or 48 h (microaerobic bacteria) The zones of bacterial growth inhibition were measured (mm) and considered as an indication of antibacterial activity.

The test determined the MIC of the extract from *N. officinale* microshoots with 3.0 mM Phe (0 day) and, for comparison, the MIC of the *N. officinale* herb extracts. Double microdilution in 96-well plates was used for the MIC test according to the CLSI method with some modifications [59]. The amount of the tested extract added to the wells with broth was such that, using the principle of double microdilution, the final concentrations were obtained in the range of 4000–62.5 µg/mL. Subsequently, each well was inoculated with 2 µL of a given bacterial strain at an inoculum density of 0.5 McFarland. On each plate there were prepared a positive control and a negative control (the broth alone), and additionally a reagent control (without bacteria—in order to rule out the error caused by the greenish color of the solutions). Next, the plates were incubated under suitable conditions for bacterial growth (aerobic bacteria at 37 °C, for 24 h; microaerobic bacteria at 37 °C, for 48 h). After incubation, microbial growth density was determined by measuring absorbance at 600 nm using a BioTek Synergy H4 (USA) automatic plate reader.

Next, the same 96-well plates were used to determine MBC; 10 µL aliquots of a mixture from all the wells that showed no visible bacterial growth were seeded onto MH/BHI agar plates. The agar plates were further incubated for 24/48 h. The lowest concentration of an extract that produced no bacterial growth was taken as its MBC. An agent is regarded as bactericidal if its MBC is no more than four times its MIC value [37]. The microbiological tests were performed in three separate experiments (*n* = 3).

### 3.8. Cytotoxicity Evaluation

The extract of *N. officinale* grown with 3.0 mM Phe (day 0) that exhibited the highest antibacterial activity was subjected to a cell culture experiment using normal human fibroblasts (BJ cell line, ATCC^®^ CRL-2522^TM^, Teddington, UK). Firstly, the BJ cells were seeded into 96-well plates at a concentration of 2 × 10^4^ cells/well. After 24 h of incubation, the culture medium was gently removed and serial dilutions of the extract from *N. officinale* grown in 3.0 mM Phe were added on day 0 (250–1.95 μg/mL) or a new portion of culture medium (0 μg/mL, control) were added. The cells were then incubated for 48 h and their viability was assessed using the MTT assay, as described previously [60].

### 3.9. Statistical Analysis

The influence of precursors and vegetation period condition was evaluated by two-way ANOVA. Cytotoxicity was evaluated using a one-way ANOVA. Differences between means were calculated using Tukey’s multiple comparison test (*p* < 0.05), using the statistical package STATISTICA 13.0 (Stat-Soft, Inc., Tulusa, OK, USA). Singular value decomposition (SVD) with imputation was used to calculate principal components (http://biit.cs.ut.ee/clustvis/). The values show the means ± SD (standard deviation). Samples were measured in three replicates.

## 4. Conclusions

These comprehensive studies have, for the first time, confirmed the impact of Phe and Trp on the production of the following metabolites: soluble saccharides, photosynthetic pigments, GSLs, flavonoids, and polyphenols in *N. officinale* microshoot cultures grown in high-production Plantform bioreactors. Our research has confirmed that the addition of the precursors at the beginning of experiments (day 0), increased the production of GSLs. Excellent stimulating effects on GSL production have been obtained for microshoot cultures grown on media with 3 mM Phe. Additionally, Phe and Trp feeding increased the production of total flavonoids (maximum for 3.0 mM Phe), polyphenols (maximum for 3.0 mM Trp), soluble saccharides (maximum for 0.05 mM Trp), and photosynthetic pigments (maximum for 0.1 mM Phe, 1 and 3 mM Trp). Moreover, the stimulating effect on the production of the following individual polyphenol compounds: *p*-coumaric acid (0.5 mM Trp), ferulic acid (3.0 mM Phe), and rutoside (0.1 mM Trp) was confirmed too.

The estimations of the antioxidant potential of the tested microshoot extracts showed that precursor feeding resulted in changes in their power.

The Phe and Trp feeding did not adversely impact biomass growth; some of the variants even stimulated microshoot multiplication. The Gi values reached by cultures maintained in the large-scale Plantform bioreactors were very high. The high biomass production and metabolite accumulation caused by the precursor-boosted *N. officinale* microshoot treatments have given us very promising results in terms of the productivity rates of secondary metabolites. This is important, especially for GSLs, which are the most important group of secondary metabolites for the species studied. The microshoot cultures were characterized by high productivity for 4-methoxyglucobrassicin and gluconasturtiin.

Based on the biomass growth, high metabolite production, and antioxidant power, the optimum conditions were chosen from the variants tested. They included the use of Phe as a precursor at a concentration of 3.0 mM (day 0). The antimicrobial activity of the biomass extracts from that variant was assessed, and the best results were proven against *P. acnes* strains, which are involved in skin diseases. What is important, our “Phe-stimulated” biomass extract was not cytotoxic to fibroblasts over the entire concentration range studied (CC_50_ value was higher than 250 μg/mL). It, therefore, promises further application research.

## Figures and Tables

**Table 1:** Growth index (Gi ± SD) values reached by bioreactor-grown *N. officinale* microshoot cultures after precursor feeding.

Day of Supplementation	C	Precursor Concentrations (mM)				
		0.05	0.1	0.5	1.0	3.0
**Phe**						
0	12.23 ^ab^ ± 1.12	11.46 ^ab^ ± 1.20	11.88 ^abcd^ ± 1.06	10.80 ^abcd^ ± 0.78	10.93 ^abcd^ ± 0.86	10.74 ^e^ ± 0.61
10	8.32 ^bcde^ ± 0.52	8.06 ^bcde^ ± 0.67	8.57 ^bcde^ ± 0.44	6.92 ^cde^ ± 0.06	10.63 ^abcd^ ± 0.89	11.48 ^abc^ ± 0.85
**Trp**						
0	12.23 ^ab^ ± 1.12	11.92 ^ab^ ± 1.00	12.46 ^ab^ ± 0.81	11.68 ^ab^ ± 0.91	12.10 ^ab^ ± 1.01	5.47 ^e^ ± 0.61
10	8.32 ^bcde^ ± 0.52	7.71 ^bcde^ ± 0.55	12.35 ^ab^ ± 1.12	8.45 ^bcde^ ± 0.32	6.28 ^de^ ± 0.20	13.66 ^a^ ± 0.77

C—control microshoots. Different superscript letters (a, b, c, etc.) indicate significant differences between means (Tukey’s test; *p* < 0.05).

**Table 2:** Total amounts of soluble saccharides (g of GLU/100 g DW ± SD) in bioreactor-grown *N. officinale* microshoot cultures after precursor feeding.

Day of Supplementation	C	Precursor Concentration (mM)				
		0.05	0.1	0.5	1.0	3.0
**Phe**						
0	4.65 ^c^ ± 0.45	4.55 ^c^ ± 0.22	6.84 ^a^ ± 1.05	5.82 ^ab^ ± 0.23	4.88 ^c^ ± 0.05	4.34 ^cd^ ± 0.01
10	5.57 ^abc^ ± 0.06	5.12 ^c^ ± 0.40	6.19 ^ab^ ± 0.01	6.01 ^ab^ ± 0.03	4.40 ^cd^ ± 0.10	4.60 ^c^ ± 0.20
**Trp**						
0	4.65 ^c^ ± 0.45	6.92 ^a^ ± 1.07	6.65 ^a^ ± 0.44	5.49 ^abc^ ± 0.02	5.60 ^ab^ ± 1.04	3.49 ^d^ ± 0.35
10	5.57 ^abc^ ± 0.06	1.58 ^e^ ± 0.22	3.91 ^cd^ ± 0.12	2.44 ^e^ ± 0.14	2.65 ^e^ ± 0.11	6.41 ^a^ ± 0.33

C—control microshoots. Different superscript letters (a, b, c, etc.) indicate significant differences between means (Tukey’s test; *p* < 0.05).

**Table 3:** Amounts of photosynthetic pigments (mg/100 g DW ± SD) in bioreactor-grown *N. officinale* microshoot cultures after precursor feeding.

Photosynthetic Pigments	Day of Supplementation	C	Precursor Concentration (mM)				
			0.05	0.1	0.5	1.0	3.0
Chlorophyll *a*	**Phe**						
	0	64.36 ^fgh^ ± 21.92	116.61 ^ab^ ± 4.73	133.32 ^a^ ± 24.93	132.57 ^a^ ± 41.47	89.82 ^cdf^ ± 17.03	118.17 ^ab^ ± 2.85
	10	50.93 ^gh^ ± 6.52	35.49 ^h^ ± 5.05	80.14 ^dfg^ ± 7.58	72.42 ^fgh^ ± 5.46	64.82 ^fgh^ ± 1.39	64.24 ^fgh^ ± 9.04
	**Trp**						
	0	64.36 ^fgh^ ± 21.92	119.32 ^ab^ ± 11.81	114.48 ^ab^ ± 16.21	118.34 ^ab^ ± 15.81	140.18 ^a^ ± 20.45	88.09 ^cdf^ ± 19.31
	10	50.93 ^gh^ ± 6.52	98.58 ^bcd^ ± 5.30	59.63 ^fgh^ ± 3.67	88.44 ^cdf^ ± 2.53	98.18 ^bcd^ ± 1.14	109.01 ^abc^ ± 12.87
Chlorophyll *b*	**Phe**						
	0	77.03 ^bcd^ ± 6.12	59.00 ^bcde^ ± 4.73	58.02 ^bcde^ ± 10.02	74.38 ^bcd^ ± 6.60	56.64 ^bcde^ ± 18.33	60.84 ^bcde^ ± 4.40
	10	38.49 ^de^ ± 5.21	29.15 ± 4.07 ^e^	71.15 ^bcd^ ± 8.23	53.75 ^cde^ ± 1.39	33.36 ^de^ ± 0.73	42.63 ^cde^ ± 6.36
	**Trp**						
	0	77.03 ^bcd^ ± 6.12	61.19 ^bcde^ ± 6.68	56.98 ^bcde^ ± 6.76	58.82 ^bcde^ ± 11.33	82.39 ^bc^ ± 2.34	148.24 ^a^ ± 5.55
	10	38.49 ^de^ ± 5.21	100.71 ^b^ ± 17.93	50.18 ^cde^ ± 3.50	53.29 ^cde^ ± 3.99	60.84 ^bcde^ ± 0.98	60.21 ^bcd^ ± 7.09
Chlorophyll *a* + *b*	**Phe**						
	0	141.39 ^cdef^ ± 8.31	175.61 ^abcde^ ± 9.45	191.28 ^abcd^ ± 34.87	207.01 ^abc^ ± 48.15	146.46 ^cdef^ ± 35.36	179.01 ^abcde^ ± 1.55
	10	89.42 ^ef^ ± 11.73	64.70 ^f^ ± 9.04	151.24 ^bcde^ ± 0.57	126.18 ^def^ ± 6.84	98.23 ^ef^ ± 0.57	106.88 ^def^ ± 15.40
	**Trp**						
	0	141.39 ^cdef^ ± 8.31	180.51 ^abcd^ ± 18.50	171.46 ^abcde^ ± 22.98	177.17 ^abcde^ ± 27.13	222.62 ^ab^ ± 43.84	236.39 ^a^ ± 74.88
	10	89.42 ^ef^ ± 11.73	199.29 ^abc^ ± 12.63	109.70 ^def^ ± 0.16	141.67 ^bcde^ ± 6.60	159.02 ^bcde^ ± 2.12	169.16 ^abcde^ ± 19.88
Carotenoids	**Phe**						
	0	3.23 ^de^ ± 0.01	20.80 ^ab^ ± 0.24	21.72 ^a^ ± 0.90	12.27 ^c^ ± 1.71	11.75 ^c^ ± 0.33	19.01 ^ab^ ± 2.44
	10	7.37 ^d^ ± 0.98	4.32 ^de^ ± 0.73	1.96 ^e^ ± 0.27	9.22 ^cd^ ± 1.75	10.72 ^cd^ ± 0.65	9.33 ^cd^ ± 1.14
	**Trp**						
	0	3.23 ^de^ ± 0.01	17.75 ^ab^ ± 1.47	18.21 ^ab^ ± 3.26	18.67 ^ab^ ± 1.79	17.80 ^ab^ ± 3.01	nd
	10	7.37 ^d^ ± 0.98	nd	4.55 ^de^ ± 0.20	9.56 ^cd^ ± 1.14	12.62 ^c^ ± 0.08	16.94 ^b^ ± 2.28

C—control microshoots. nd—not detected. Different superscript letters (a, b, c, etc.) indicate significant differences between means (Tukey’s test; *p* < 0.05).

**Table 4:** Total amounts of GSLs (mg SIN/100 g DW ± SD), flavonoids (mg RE/100 g DW ± SD), and polyphenols (mg GAL/100 g DW ± SD) in bioreactor-grown *N. officinale* microshoot cultures after precursor feeding.

Method	Day of Supplementation	C	Precursor Concentration (mM)				
			0.05	0.1	0.5	1.0	3.0
Total GSLs content	**Phe**						
	0	81.58 ^c^ ± 2.18	181.10 ^abc^ ± 11.50	163.51 ^abc^ ± 20.78	141.06 ^bc^ ± 10.65	147.48 ^bc^ ± 20.68	269.20 ^a^ ± 15.78
	10	125.04 ^bc^ ± 5.05	180.07 ^abc^ ± 15.73	142.89 ^bc^ ± 17.63	141.40 ^bc^ ± 15.66	196.73 ^ab^ ± 20.60	216.82 ^ab^ ± 18.87
	**Trp**						
	0	81.58 ^c^ ± 2.18	200.44 ^ab^ ± 12.04	194.89 ^ab^ ± 23.76	187.84 ± 27.11	184.27 ^ab^ ± 28.43	196.26 ^ab^ ± 12.56
	10	125.04 ^bc^ ± 5.05	190.72 ^ab^ ± 20.56	168.46 ^abc^ ± 28.34	140.09 ^abc^ ± 20.40	200.97 ^ab^ ± 5.17	161.70 ^abc^ ± 10.91
Total flavono-ids content	**Phe**						
	0	565.16 ^g^ ± 14.32	1124.60 ^bcd^ ± 102.47	1211.09 ^bc^ ± 24.31	1188.98 ^bcd^ ± 39.50	1118.99 ^bcd^ ± 51.65	1364.38 ^a^ ± 80.14
	10	863.71 ^fe^ ± 49.96	808.38 ^fe^ ± 45.35	1043.81 ^cdf^ ± 5.68	944.88 ^dfe^ ± 9.07	878.70 ^fe^ ± 21.79	1016.75 ^cdf^ ± 23.76
	**Trp**						
	0	565.16 ^g^ ± 14.32	965.82 ^dfe^ ± 11.02	1032.24 ^cdf^ ± 91.49	958.72 ^dfe^ ± 16.55	1324.14 ^ab^ ± 123.67	1241.89 ^abc^ ± 74.62
	10	863.71 ^fe^ ± 49.96	739.22 ^f^ ± 55.75	825.89 ^fe^ ± 33.91	1131.38 ^bcd^ ± 120.72	1169.84 ^bcd^ ± 79.37	964.93 ^dfe^ ± 142.40
Total poly-phenols content	**Phe**						
	0	189.61 ^i^ ± 25.82	251.37 ^defg^ ± 15.50	325.55 ^c^ ± 7.99	266.47 ^def^ ± 9.46	253.55 ^defg^ ± 9.90	282.68 ^d^ ± 7.75
	10	248.02 ^efg^ ± 4.55	235.10 ^fgh^ ± 8.99	228.29 ^gh^ ± 9.34	237.24 ^fg^ ± 0.05	274.53 ^de^ ± 3.98	244.28 ^efg^ ± 9.20
	**Trp**						
	0	189.61 ^i^ ± 25.82	258.81 ^dfg^ ± 5.05	258.59 ^dfg^ ± 10.01	271.26 ^de^ ± 15.39	349.34 ^c^ ± 15.56	873.11 ^b^ ± 40.89
	10	248.02 ^efg^ ± 4.55	151.43 ^j^ ± 14.05	201.89 ^hi^ ± 7.90	243.18 ^efg^ ± 10.06	335.12 ^c^ ± 7.94	1062.76 ^a^ ± 28.77

C—control microshoots. Different superscript letters (a, b, c, etc.) indicate significant differences between means (Tukey’s test; *p* < 0.05).

**Table 5:** The UHPLC-DAD-MS/MS GSLs profile and productivity of bioreactor-grown *N. officinale* microshoot cultures after precursor feeding.

Subgroups of GSLs	No. *	GSLs (Trivial Name)	*t*_R_ (min)	[M + Na]^+^	C		Precursor Treatments			
							3.0 mM Phe, Day 0		3.0 mM Trp, Day 0	
					GSLs mg/100 g DW ± SD	Productivity	GSLs mg/100 g DW ± SD	Productivity	GSLs mg/100 g DW ± SD	Productivity
Methionine derived	1	7-(Methylsulfinyl)heptyl GSL	6.55	422	tr	nd	tr	nd	nd	nd
	2	8-(Methylsulfinyl)octyl GSL (Glucohirsutin)	7.58	436	tr	nd	tr	nd	nd	nd
Phenylalanine derived	3	2-Phenylethyl GSL (Gluconasturtiin)	8.20	366	15.65 ± 1.49	35.77	36.71 ± 3.32	97.59	8.83 ± 0.89	13.14
Tryptophan derived	4	4-Hydroxyindol-3-ylmethyl GSL (Hydroxyglucobrassicin)	5.85	407	nd	nd	tr	nd	tr	nd
	5	Indol-3-ylmethyl GSL (Glucobrassicin)	7.64	391	tr	nd	6.78 ± 0.04	18.03	3.78 ± 0.24	5.63
	6	4-Methoxyindol-3-ylmethyl GSL (4-Methoxyglucobrassicin)	8.35	421	12.79 ± 1.03	29.23	149.99 ± 17.44	398.77	76.12 ± 3.12	113.31

C—control microshoots, [M + Na]^+^—sodium adduct of desulfoglucosinolate; tr—<0.1 μmol/g DW; nd—not detected. Productivity = $\frac{\max.\mathrm{concentration}\;\mathrm{for}\;\mathrm{each}\;\mathrm{molecule}\;\mathrm{in}\;\mathrm{mg}/100\;g\;\mathrm{DW}\times \mathrm{culture}\;\mathrm{biomass}\;\mathrm{in}\;g/L}{100\;g\;\mathrm{DW}}$. * Table 5 is corresponding to Figure S2.

**Table 6:** Amounts of individual polyphenol compounds (mg/100 g DW ± SD) in extracts of bioreactor-grown *N. officinale* microshoot cultures after precursor feeding.

Polyphenol Compound	Day of Supplementation	C	Precursor Concentrations (mM)				
			0.05	0.1	0.5	1.0	3.0
*p*-Coumaric acid	**Phe**						
	0	10.99 ^c^ ± 2.07	13.54 ^c^ ± 0.82	16.27 ^bc^ ± 1.32	13.68 ^c^ ± 2.58	13.71 ^c^ ± 3.32	23.38 ^ab^ ± 3.55
	10	4.90 ^e^ ± 0.35	8.74 ^d^ ± 0.02	11.39 ^c^ ± 1.01	10.20 ^c^ ± 1.06	25.38 ^a^ ± 2.11	12.19 ^c^ ± 1.34
	**Trp**						
	0	10.99 ^c^ ± 2.07	22.57 ^b^ ± 3.35	13.79 ^c^ ± 0.09	14.05 ^c^ ± 1.22	2.51 ^e^ ± 0.20	15.09 ^bc^ ± 0.33
	10	4.90 ^e^ ± 0.35	21.54 ^b^ ± 0.42	24.65 ^a^ ± 2.64	29.11 ^a^ ± 3.70	8.97^d^ ± 0.92	24.97 ^a^ ± 5.87
Ferulic acid	**Phe**						
	0	3.66 ^d^ ± 0.70	9.58 ^cd^ ± 0.27	13.64 ^c^ ± 0.82	10.84 ^cd^ ± 2.44	18.20 ^b^ ± 3.20	27.76 ^a^ ± 2.12
	10	2.62 ^e^ ± 0.35	2.90 ^e^ ± 0.26	2.87 ^e^ ± 0.11	4.98 ^d^ ± 0.07	13.04 ^c^ ± 0.62	24.30 ^a^ ± 0.79
	**Trp**						
	0	3.66 ^d^ ± 0.70	13.70 ^c^ ± 2.07	6.45 ^d^ ± 0.11	6.34 ^d^ ± 0.59	4.35^d^ ± 0.08	8.47 ^cd^ ± 0.88
	10	2.62 ^e^ ± 0.35	9.75 ^cd^ ± 0.30	18.12 ^b^ ± 1.43	19.64 ^b^ ± 1.91	6.07 ^d^ ± 0.54	13.81 ^c^ ± 1.98
Rutoside	**Phe**						
	0	3.82 ^d^ ± 0.60	5.18 ^cd^ ± 0.36	6.71 ^b^ ± 0.90	6.57 ^c^ ± 0.36	1.26 ^e^ ± 0.32	9.50 ^b^ ± 1.14
	10	2.94 ^d^ ± 0.52	4.02 ^d^ ± 0.02	5.23 ^cd^ ± 0.06	5.25 ^cd^ ± 0.13	5.30 ^cd^ ± 0.09	9.94 ^b^ ± 0.89
	**Trp**						
	0	3.82 ^d^ ± 0.60	11.66 ^b^ ± 1.44	3.89 ^d^ ± 0.07	3.34 ^d^ ± 0.59	2.81 ^d^ ± 0.04	3.51 ^d^ ± 0.67
	10	2.94 ^d^ ± 0.52	2.48 ^d^ ± 0.12	16.03 ^a^ ± 1.28	11.64 ^b^ ± 1.23	3.89 ^d^ ± 0.21	7.55 ^c^ ± 0.66

C—control microshoots. Different superscript letters (a, b, c, etc.) indicate significant differences between means (Tukey’s test; *p* < 0.05).

**Table 7:** Antioxidant activity estimated using the CUPRAC, DPPH, and FRAP assays (mmol TE/100 g DW ± SD) of bioreactor-grown *N. officinale* microshoot cultures after precursor feeding.

Assay	Day of Supplementation	C	Precursor Concentrations (mM)				
			0.05	0.1	0.5	1.0	3.0
CUPRAC	**Phe**						
	0	1.54 ^j^ ± 0.06	2.61 ^b^ ± 0.14	3.05 ^a^ ± 0.18	2.51 ^bc^ ± 0.02	2.32 ^defg^ ± 0.07	3.02 ^a^ ± 0.01
	10	2.15 ^gh^ ± 0.01	1.71 ^j^ ± 0.04	2.18 ^fgh^ ± 0.01	2.27 ^defg^ ± 0.02	2.22 ^efgh^ ± 0.11	2.14 ^gh^ ± 0.03
	**Trp**						
	0	1.54 ^j^ ± 0.06	2.39 ^cde^ ± 0.02	2.34 ^def^ ± 0.01	2.37 ^de^ ± 0.01	2.70 ^b^ ± 0.09	2.09 ^h^ ± 0.02
	10	2.15 ^gh^ ± 0.01	1.90 ^i^ ± 0.16	1.70 ^j^ ± 0.07	2.45 ^cd^ ± 0.04	2.72 ^b^ ± 0.06	2.34 ^def^ ± 0.03
DPPH	**Phe**						
	0	0.47^j^ ± 0.02	0.66 ^dfgh^ ± 0.04	0.86 ^ab^ ± 0.05	0.77 ^bcd^ ± 0.05	0.77 ^bcd^ ± 0.02	0.85 ^ab^ ± 0.03
	10	0.69 ^dfg^ ± 0.09	0.73 ^bcd^ ± 0.06	0.72 ^cdf^ ± 0.07	0.57 ^hij^ ± 0.02	0.82 ^abc^ ± 0.01	0.64 ^efghi^ ± 0.06
	**Trp**						
	0	0.47^j^ ± 0.02	0.60 ^ghi^ ± 0.04	0.54 ^ij^ ± 0.02	0.64 ^efghi^ ± 0.01	0.62 ^fghi^ ± 0.09	0.69 ^dfg^ ± 0.09
	10	0.69 ^dfg^ ± 0.09	0.77 ^bcd^ ± 0.06	0.54 ^ij^ ± 0.03	0.90 ^a^ ± 0.03	0.62 ^fghi^ ± 0.01	0.61 ^fghi^ ± 0.03
FRAP	**Phe**						
	0	0.24 ^i^ ± 0.01	0.61 ^bc^ ± 0.02	0.94 ^a^ ± 0.26	0.55 ^cde^ ± 0.01	0.57 ^cd^ ± 0.01	0.73 ^b^ ± 0.03
	10	0.40 ^fgh^ ± 0.02	0.32 ^hi^ ± 0.05	0.40 ^gh^ ± 0.04	0.39 ^gh^ ± 0.01	0.52 ^cdfg^ ± 0.01	0.31 ^hi^ ± 0.11
	**Trp**						
	0	0.24 ^i^ ± 0.01	0.52 ^cdfg^ ± 0.01	0.49 ^cdfg^ ± 0.01	0.52 ^cdfg^ ± 0.01	0.53 ^cdf^ ± 0.02	0.42 ^fgh^ ± 0.01
	10	0.40 ^fgh^ ± 0.02	0.33 ^hi^ ± 0.02	0.34 ^hi^ ± 0.02	0.43 ^fgh^ ± 0.01	0.49 ^cdfg^ ± 0.03	0.44 ^dfgh^ ± 0.01

C—control microshoots. Different superscript letters (a, b, c, etc.) indicate significant differences between means (Tukey’s test; *p* < 0.05).

**Table 8:** Minimum inhibitory concentration (MIC, µg/mL) and ratio of minimum bactericidal concentration to MIC (MBC/MIC) of extracts from bioreactor-grown *N. officinale* microshoot cultures (*p* < 0.05 vs. control, *n* = 3).

*N. officinale* Extract	*S. aureus* ATCC 25923		*S. epidermidis* ATCC 12228		*P. acnes* PCM 2400		*P. acnes* PCM 2334		*P. granulosum* PCM 2462	
	MIC	$\frac{MBC}{MIC}$	MIC	$\frac{MBC}{MIC}$	MIC	$\frac{MBC}{MIC}$	MIC	$\frac{MBC}{MIC}$	MIC	$\frac{MBC}{MIC}$
C	2000	>8	2000	>8	1000	>8	1000	>8	500	16
Phe	1000	>8	1000	8	500	8	250	8	500	8

C—control microshoots not treated with precursors on day 0; Phe—microshoots treated with 3.0 mM Phe on day 0.

## Supplementary Materials

The following are available online, Figure S1: Morphological appearance of *N. officinale* agitated microshoots cultures after precursor feeding, Figure S2: Chromatogram of desulfoglucosinolates (dGSL): **1**—7-(methylsulfinyl)heptyl dGSL; **2**—8-(methylsulfinyl)octyl dGSL; **3**—desulfogluconasturtiin; **4**—4-hydroxyindol-3-ylmethyl dGSL; **5**—indol-3-ylmethyl dGSL; **6**—4-methoxyindol-3-ylmethyl dGSL, Table S1: Growth index (Gi ± SD) values reached by *N. officinale* agitated microshoots cultures after precursor feeding (*p* < 0.05 vs. control, *n* = 6), Table S2: Total amounts of GSLs (mg SIN/100 g DW ± SD) in *N. officinale* agitated microshoots cultures after precursor feeding (*p* < 0.05 vs. control, *n* = 6), Table S3: Amounts of individual polyphenol compounds (mg/100 g DW ± SD) in extracts of *N. officinale* agitated microshoots cultures after precursor feeding (*p* < 0.05 vs. control, *n* = 6), Table S4: Antioxidant activity estimated using the CUPRAC assay (mmol of TE/100 g DW ± SD) of *N. officinale* agitated microshoots cultures after precursor feeding (*p* < 0.05 vs. control, *n* = 6).

## Sample Availability

Samples of the compounds are available from the authors.
