# Supplementary material for: Precursor-Boosted Production of Metabolites in Nasturtium officinale Microshoots Grown in Plantform Bioreactors, and Antioxidant and Antimicrobial Activities of Biomass Extracts
Source: Molecules. 2021 Jul 31;26(15):4660. doi: 10.3390/molecules26154660 (PMC8348939; doi:10.3390/molecules26154660)
Supplement: Supplementary file 1 [file molecules-26-04660-s001.zip › molecules-1277803-supplementary.pdf]

Supplementary material

Precursor-Boosted Production of Metabolites in *Nasturtium officinale* Microshoots Grown in Plantform Bioreactors, and Antioxidant and Antimicrobial Activities of Biomass Extracts

Marta Klimek-Szczykutowicz [1,2], Michał Dziurka [3], Ivica Blažević [4], Azra Đulović [4], Małgorzata Miazga-Karska [5], Katarzyna Klimek [5], Halina Ekiert [1], Agnieszka Szopa [1*]

[1] Chair and Department of Pharmaceutical Botany, Faculty of Pharmacy, Jagiellonian University, Medical College, Medyczna 9, 30-688 Kraków, Poland; marta.klimek-szczykutowicz@doctoral.uj.edu.pl (M.K-S.); halina.ekiert@uj.edu.pl (H.E.)

[2] Department of Dermatology, Cosmetology and Aesthetic Surgery, The Institute of Medical Sciences, Medical College, Jan Kochanowski University, Stefana Żeromskiego 5, 25-369 Kielce, Poland

[3] The Franciszek Górski Institute of Plant Physiology, Polish Academy of Sciences, Niezapominajek 21,30-239 Kraków, Poland; m.dziurka@ifr-pan.edu.pl (M.D.)

[4] Department of Organic Chemistry, Faculty of Chemistry and Technology University of Split, Ruđera Boškovića 35, 21000 Split, Croatia; blazevic@ktf-split.hr (I.B.); azra@ktf-split.hr (A.Đ.)

[5] Chair and Department of Biochemistry and Biotechnology, Faculty of Pharmacy, Medical University of Lublin, Chodzki 1 Street, 20-046 Lublin, Poland; malgorzata.miazga-karska@umlub.pl (M.M-K.); katarzynaklimek@umlub.pl (K.K.)

* Correspondence: a.szopa@uj.edu.pl (A.S.); Tel.: +48 12 620 54 30, fax +48 620 54 40

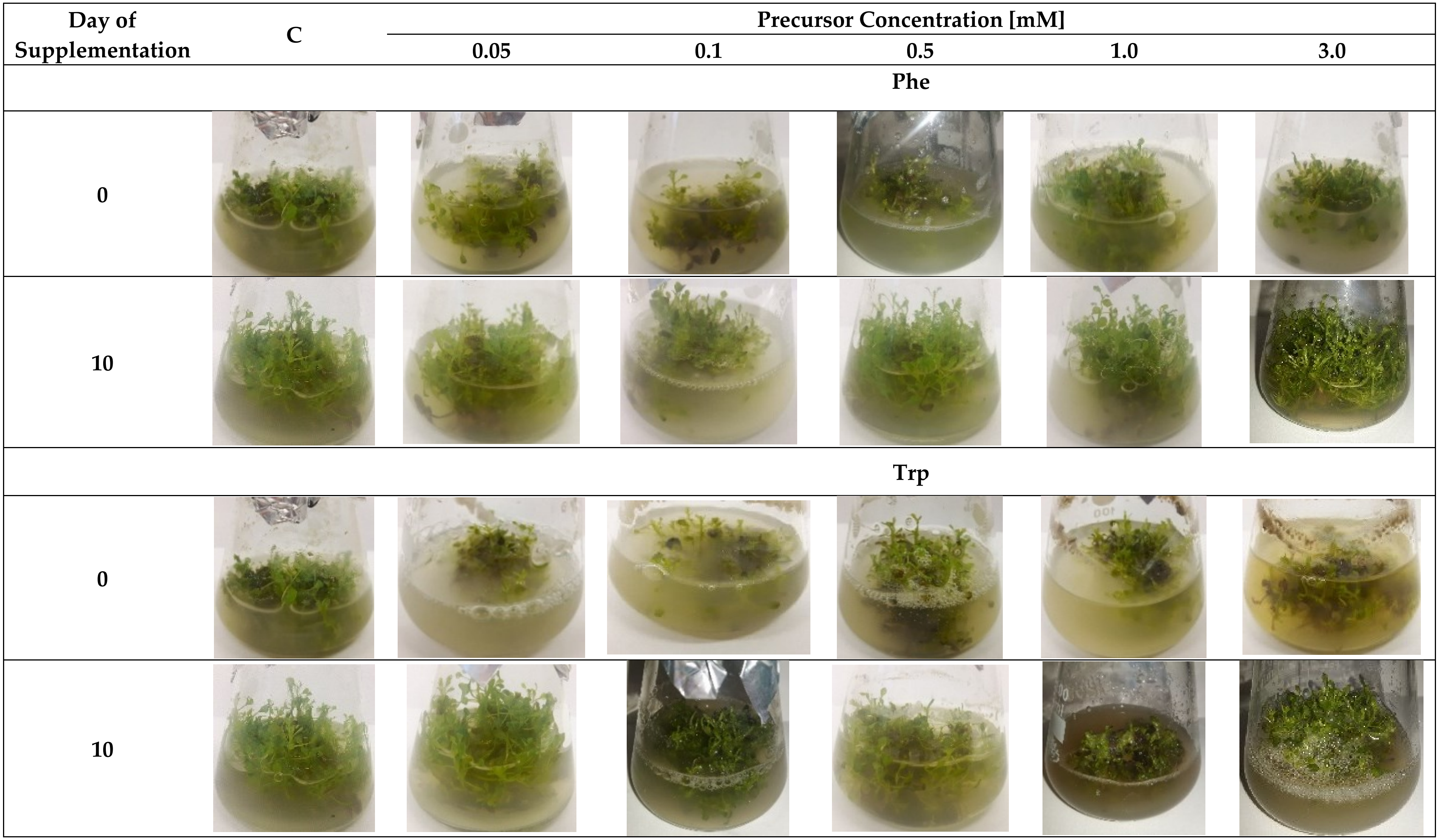

Figure S1. Morphological appearance of *N. officinale* agitated microshoots cultures after precursor feeding.

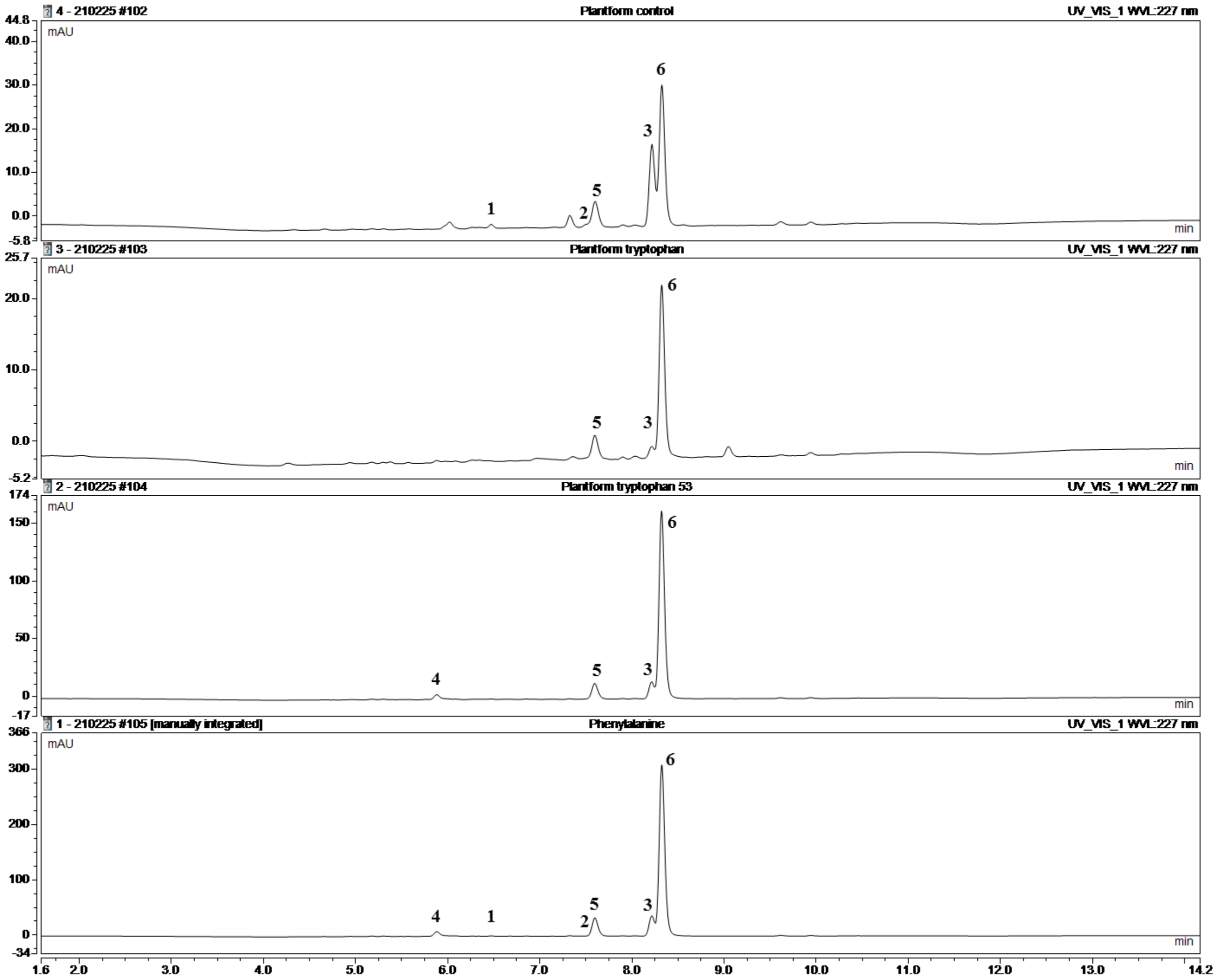

Figure S2. Chromatogram of desulfoglucosinolates (dGSL): **1** – 7-(methylsulfinyl)heptyl dGSL; **2** – 8-(methylsulfinyl)octyl dGSL; **3** – desulfogluconasturtiin; **4** – 4-hydroxyindol-3-ylmethyl dGSL; **5** – indol-3-ylmethyl dGSL; **6** – 4-methoxyindol-3-ylmethyl dGSL

Table S1. Growth index (Gi±SD) values reached by *N. officinale* agitated microshoots cultures after precursor feeding ($p < 0.05$ *vs* control, n=6).

**Day of Supplementation**	**C**	**Precursor Concentration [mM]**				
		**0.05**	**0.1**	**0.5**	**1.0**	**3.0**
				**Phe**		
**0**	8.95±0.10	5.92±0.04	8.34±0.03	8.11±0.04	7.59±0.04	10.74±0.07
**10**	8.90±0.13	7.00±0.02	8.30±0.04	7.90±0.04	8.94±0.05	8.72±0.03
				**Trp**		
**0**	8.95±0.10	3.78±0.04	3.75±0.03	4.75±0.09	5.58±0.12	6.53±0.01
**10**	8.90±0.13	8.20±0.05	13.22±0.05	7.73±0.03	10.92±0.01	8.60±0.10

Table S2. Total amounts of GSLs (mg SIN/100 g DW±SD) in *N. officinale* agitated microshoots cultures after precursor feeding ($p < 0.05$ *vs* control, n=6).

Day of Supplementation	C	Precursor Concentration [mM]				
		**0.05**	**0.1**	**0.5**	**1.0**	**3.0**
				**Phe**		
**0**	202.75±23.01	321.04±15.33	324.73±18.00	280.41±15.05	293.92±20.60	159.44±20.50
**10**	179.37±26.90	227.80±21.45	300.31±16.76	283.89±2.58	238.19±20.10	214.54±19.50
				**Trp**		
**0**	202.75±23.01	290.05±20.10	276.39±15.80	245.72±10.11	273.86±15.23	318.40±20.33
**10**	179.37±26.90	285.79±20.26	206.10±24.01	294.25±28.83	199.08±9.86	165.76±26.92

Table S3. Amounts of individual polyphenol compounds (mg/100g DW±SD) in extracts of *N. officinale* agitated microshoots cultures after precursor feeding ($p < 0.05$ *vs* control, n=6).

Polyphenol Compound	Day of Supplementation	C	Precursor Concentration (mM)				
			0.05	0.1	0.5	1.0	3.0
					Phe		
p-Coumaric acid	0	10.07±0.99	12.53±1.32	37.17±3.98	21.12±2.55	24.79±3.09	28.40±3.11
	10	7.11±0.69	20.24±0.21	28.49±0.33	12.33±0.21	19.57±0.60	16.68±0.33
					Trp		
	0	10.07±0.99	28.49±3.67	25.28±3.01	20.92±2.66	47.44±5.12	40.28±3.98
	10	7.11±0.69	11.01±1.00	52.20±5.12	22.29±1.42	12.67±1.32	26.36±3.45
Ferulic acid					Phe		
	0	7.29±0.68	6.66±0.71	19.38±2.00	12.79±1.99	16.51±6.70	18.50±2.13
	10	0.95±0.09	7.36±0.02	14.75±0.16	6.05±0.05	7.02±0.34	10.65±1.22
					Trp		
	0	7.29±0.68	10.34±0.89	10.94±0.94	9.69±0.87	13.44±1.53	14.64±1.65
	10	0.95±0.09	6.63±0.59	45.34±4.53	11.00±1.08	7.37±0.67	25.09±2.12
Rutoside					Phe		
	0	2.22±0.19	3.19±0.44	13.10±1.41	4.36±0.39	3.90±0.30	8.91±0.77
	10	0.73±0.12	2.85±0.01	7.36±0.08	2.06±0.01	2.69±0.21	6.27±0.70
					Trp		
	0	2.22±0.19	2.57±0.34	4.45±0.52	4.67±0.51	7.00±0.65	25.52±3.09
	10	0.73±0.12	1.33±0.01	44.05±3.89	10.11±1.02	4.47±0.51	6.55±0.77

Table S4. Antioxidant activity estimated by the CUPRAC assay (mmol of TE/100 g DW±SD) of *N. officinale* agitated microshoots cultures after precursor feeding ($p < 0.05$ *vs* control, n=6).

Day of Supplementation	C	Precursor Concentration [mM]				
		**0.05**	**0.1**	**0.5**	**1.0**	**3.0**
				**Phe**		
**0**	3.54±0.10	2.83±0.06	2.78±0.15	2.34±0.78	3.19±0.09	3.52±0.31
**10**	2.07±0.52	2.55±0.48	2.87±0.32	2.97±0.17	3.40±0.05	3.45±0.06
				**Trp**		
**0**	3.54±0.10	3.09±0.32	3.34±0.08	3.59±0.10	3.55±0.09	3.36±0.07
**10**	2.07±0.52	2.70±0.22	3.54±0.11	2.77±0.06	2.74±0.15	2.72±0.14
